# Interplay of Adiponectin With Glycemic and Metabolic Risk Metrics in Patients With Diabetes

**DOI:** 10.7759/cureus.70543

**Published:** 2024-09-30

**Authors:** Ritu Tiwari, Nishtha Singh, Shraddha Singh, Manish Bajpai, Shivam Verma

**Affiliations:** 1 Physiology, King George's Medical University, Lucknow, IND; 2 Microbiology, T.S. Misra Medical College and Hospital, Lucknow, IND; 3 Physiology, Prasad Institute of Medical Sciences, Lucknow, IND

**Keywords:** adiponectin, bmi, glycemic control, lipid profile, t2dm

## Abstract

Background

Type 2 diabetes mellitus (T2DM) is characterized by low insulin production or resistance. Adiponectin, a hormone produced by adipocytes, is essential for regulating glucose metabolism and is correlated with insulin decrease.

Aim

The aim of this study is to estimate the levels of adiponectin in T2DM patients and their relationship with various metabolic markers (glycated hemoglobin (HbA1c), fasting blood sugar (FBS), fasting insulin, lipid profile, and anthropometric variables in an Indian cohort.

Methods

This study was conducted at King George’s Medical University (KGMU), India, from October 2022 to October 2024. The study (case-control) included 234 subjects: T2DM patients and age-sex-matched healthy controls. Diagnosis of T2DM followed American Diabetes Association criteria. Data collection involved medical history, anthropometric measurements, blood pressure, and biochemical assessments including FBS, HbA1c, lipid profiles, insulin levels, and insulin resistance. Serum adiponectin levels were estimated using an ELISA kit.

Results

T2DM patients had a significantly higher HbA1c value (7.82±0.96%) compared to controls (5.31±0.39%, p<0.001). Insulin resistance was also significantly higher in T2DM patients (4.31±0.95) than in controls (3.62±0.82, p=0.002). Adiponectin levels were significantly lower in T2DM patients (6.87±3.73 µg/mL) compared to controls (10.18±5.16 µg/mL, p<0.001). Low levels of adiponectin were correlated with HbA1c (r=-0.58, p<0.001), FBS (r=-0.51, p<0.001), and total cholesterol (r=0.38, p<0.001). Adiponectin levels also were correlated with BMI (r=-0.33, p<0.001).

Conclusion

Lower adiponectin levels in T2DM patients correlate with HbA1c and increased insulin resistance, suggesting that adiponectin may be a biomarker for diabetes management and risk assessment.

## Introduction

Type 2 diabetes mellitus (T2DM) is a prevalent metabolic syndrome characterized by chronic hyperglycemia after either inadequate insulin production, insulin resistance, or both [1]. T2DM has dramatically risen in India over recent decades. International Diabetes Federation (IDF) data revealed that in India 77 million people with T2DM in 2019, and projections estimate that this will increase to 134 million by 2045 [2,3]. This surge is attributed to significant lifestyle changes, including decreased physical activity and increased consumption of processed, high-calorie foods such as refined carbohydrates, saturated fats, and sugars, which exacerbate insulin impairment and contribute to the development of T2DM [4,5].

Adiponectin, an insulin-sensitizing hormone secreted by adipocytes or fat cells, plays an important role in regulating insulin sensitivity and glucose metabolism [6]. It is involved in various metabolic processes relevant to T2DM, including glucose homeostasis, insulin sensitivity, lipid, and inflammation [7]. Adiponectin enhances fatty acid oxidation and improves insulin receptor substrate (IRS) signaling in the liver and skeletal muscles, which facilitates better glucose metabolism [8,9]. Low levels of adiponectin are observed in individuals with T2DM, which correlates with high HbA1c and an increased risk of cardiovascular disease [10].

The role of adiponectin in metabolic syndrome is well studied in Western populations; however, there is a notable lack of studies focusing on its relevance within the Indian subcontinent context. Most studies have primarily investigated the relationship between adiponectin and various metabolic markers in Western cohorts, leaving a gap in understanding how these associations might differ in Indian populations [11]. Specifically, there is limited data on adiponectin levels in relation to FBS, HbA1c, lipid profiles, and anthropometric indicators of cardiometabolic risk in India.

This study seeks to address this gap by evaluating adiponectin levels in T2DM patients and exploring their relationships with glycemic control, lipid profiles, and cardiometabolic risk factors in the Indian subcontinent (Lucknow and nearby district, Uttar Pradesh), by comparing these findings with age-matched healthy controls.

## Materials and methods

Study population and selection criteria

This case-control study involved T2DM patients (n=117) and age and sex-matched normal controls (n=117). This study was conducted from October 2022 to May 2024 at the Department of Physiology in collaboration with the Departments of Medicine and Biochemistry at King George’s Medical University (KGMU), Lucknow, India.

The case group consisted of individuals aged 20-70 years diagnosed with T2DM based on criteria from the American Diabetes Association (ADA): glycated hemoglobin (HbA1c) level of ≥ 6.5%, fasting blood sugar (FBS) level of ≥ 126, or a random plasma glucose level ≥ 200 mg/dL in the presence of diabetes symptoms [1].

Inclusion and exclusion criteria

Patients with diabetes with other chronic diseases and control participants were matched for age and sex, who visited for routine check-ups, and no chronic illnesses were included.

Exclusions were made for type 1 diabetes, anatomical deformities affecting measurements, pregnancy, chronic conditions (such as thyroid disorders, renal diseases, autoimmune disorders, and cancer), substance abuse, and glucocorticoid therapy.

Ethical consideration

The study was approved by the institutional ethical committee (Ref. code: 125th ECMIIB-Ph.D/P1)KGMU, and written informed consent was obtained from all participants.

Data collection

Demographics, with a history of body mass index (BMI), waist circumference (WC), blood pressure (BP), and pulse rate (PR), were obtained. Skinfold thickness measurements were taken using Harpenden Skinfold Calipers, precise to the nearest 0.1 mm, while neck circumference was recorded 0.1 cm below the laryngeal prominence using a flexible tape measure [12]. 

Biochemical assessments

Fasting blood samples were collected from participants after 10-12 hours of fasting. An automated random access biochemistry analyzer (Cobas 6000, Roche Diagnostics) measured fasting glucose, lipid profiles, and insulin levels. HbA1c was measured using the BIO-RAD Variant II Hemoglobin Testing System (Bio-Rad Laboratories, Hercules, US). The homeostatic model assessment for insulin resistance (HOMA-IR) was calculated using the formula: “Fasting insulin (µU/L) X Fasting glucose (nmol/L)/22.5” [13].

Estimation of circulating adiponectin levels

A sandwich enzyme-linked immunosorbent assay (ELISA) was used to measure the serum levels of adiponectin as recommended by the manufacturer protocol (Human ADP/Acrp30, Kit, Cat No. E-EL-H6122, Elabscience, USA). 

Data analysis

The data were analyzed using IBM SPSS Statistics for Windows, Version 21 (Released 2012; IBM Corp., Armonk, New York, United States). The descriptive statistics were presented in terms of frequencies (percent) and the mean ± standard deviation (SD). Statistical analysis was conducted using Student's t-test and the chi-square test. An assessment of the relationship between the variables was performed using the Pearson correlation coefficient. A significance level of p< 0.05 was taken to be statistically significant.

## Results

Status of glycemic markers and lipid profiles in T2DM

Age (50.22±16.76 vs. 46.76±19.45 years, p=0.146) and gender distribution for T2DM group vs control group (male 66 (56.4%) vs. male 62 (53.0%), p=0.69 respectively) showed non-significant differences between groups, indicating an adequate match.

HbA1c levels were significantly higher in the T2DM group (7.82±0.96%) than in the controls (5.31±0.39%, p<0.001). Similarly, FBS was significantly (p<0.001) higher in the T2DM group (125.21±14.64) than in the control group (93.64±7.23 mg/dL). Insulin levels were also increased in the T2DM group (15.41±7.43) than in controls (13.10±3.42 IU, p=0.002). Insulin resistance (4.31±0.95 vs. 3.62±0.82, p=0.002) was significantly higher in T2DM group. Total cholesterol (TC) levels were high and HDL levels were low in the T2DM group (203.65±31.96 and 35.80±6.61 mg/dL, respectively) compared to the control group (169.09±23.14, p=0.005 and 29.49±4.70 mg/dL, p=0.006 respectively). VLDL levels were significantly elevated in the diabetes group (38.16±6.84 mg/dL) compared to controls (20.90±4.02 mg/dL, p<0.001).

Adiponectin levels were significantly lower (6.87±3.73) in the T2DM group than in the control group (10.18±5.16 µg/mL, p<0.001). TG (195.31±43.85 vs. 106.42±38.33 mg/dL, p=0.07) and LDL (136.29±31.05 vs. 111.90±25.62 mg/dL, p=0.13) did not show significant differences for both the groups (Table 1).

**Table 1:** Comparison of biochemical variables in diabetic patients and normal control HOMA-IR: Homeostatic Model Assessment for Insulin Resistance; TC: Total cholesterol; TG: Triglyceride; HDL: High-density lipoprotein; LDL: Low-density lipoprotein; VLDL: Very low-density lipoprotein. Student's t-test was used to calculate the p-value. *p-value <0.05 was considered as statistically significant.

Variables	Diabetes (n=117) Mean±SD	Control (n=117) Mean±SD	p-value	Chi-square
Gender: Male N (%) Female N (%)	66(56.4); 51(43.6)	62(53.0); 55(47.0)	0.59	0.27
Age (years); range	50.22±16.76; 42-59	46.76±19.45; 39-51	0.15	1.45
HbA1c (%)	7.82±0.96	5.31±0.39	<0.0001*	26.20
FBS (mg/dL)	125.21±14.64	93.64±7.23	<0.0001*	20.91
Insulin (IU)	15.41±7.43	13.10±3.42	0.002*	3.05
HOMA-IR	4.31±0.95	3.62±0.82	0.002*	5.94
TC (mg/dL)	203.65±31.96	169.09±23.14	0.005*	9.47
TG (mg/dL)	195.31±43.85	106.42±38.33	0.07*	16.51
HDL (mg/dL)	29.49±4.70	35.80±6.61	0.006*	8.41
LDL (mg/dL)	136.29±31.05	111.90±25.62	0.13	6.55
VLDL (mg/dL)	38.16±6.84	20.90±4.02	<0.0001*	23.53
Adiponectin (µg/mL)	6.87±3.73	10.18±5.16	<0.0001*	-5.62

Cardiovascular and anthropometric status in T2DM patients

The T2DM group showed higher systolic blood pressure (138.55±6.53 vs. 122.43±9.24 mmHg, p<0.001) and diastolic blood pressure (91.39±6.86 vs. 74.59±8.59 mmHg, p=0.003) than the controls. The T2DM group had a significantly higher NC (36.89±2.84 vs. 35.21±3.28 cm, p=0.01) and hip circumference (HC) (97.13±3.56 vs. 83.47±12.19 cm, p<0.001). Furthermore, there was a slightly increased but non-significant trend was found for weight (81.79±11.54 vs. 72.30±10.76 kg, p=0.669), height (167.24±6.81 vs. 167.49±7.38 cm, p=0.772), BMI (26.25±3.89 vs. 25.62±2.09,p=0.124), AC (89.21±8.33 vs. 88.17±9.09 cm, p=0.253), WC (93.62±9.08 vs. 92.11±8.10 cm, p=0.057), and HC (97.13±3.56 vs. 83.47±12.19, p<0.001) (Table 2).

**Table 2:** Status of anthropometric parameters in diabetic patients and normal control BMI: Body mass index, AC: Abdominal circumference, NC: Neck circumference, HC: Hip circumference. Student's t-test was used to calculate the p-value. *p<0.05 was considered statistically significant.

Variables	Diabetic (n=117) Mean±SD	Control (n=117) Mean±SD	p-value	t-test
Systolic (mmHg)	138.55±6.53	122.43±9.24	<0.0001*	15.41
Diastolic (mmHg)	91.39±6.86	74.59±8.59	0.003*	16.53
Weight (kg)	81.79±11.54	72.30±10.76	0.669	6.50
Height (cm)	167.24±6.81	167.49±6.38	0.772	-0.29
BMI (kg/m^2^)	26.25±3.89	25.62±2.09	0.124	1.54
AC (cm)	89.21±8.33	88.17±9.09	0.253	0.91
NC (cm)	36.89±2.84	35.21±3.28	0.010*	4.18
WC (cm)	93.62±9.08	92.11±8.10	0.057	1.34
HC (cm)	97.13±3.56	83.47±12.19	<0.0001*	11.63

Adiponectin correlated with glycemic markers and lipid profile

**Figure 1:**
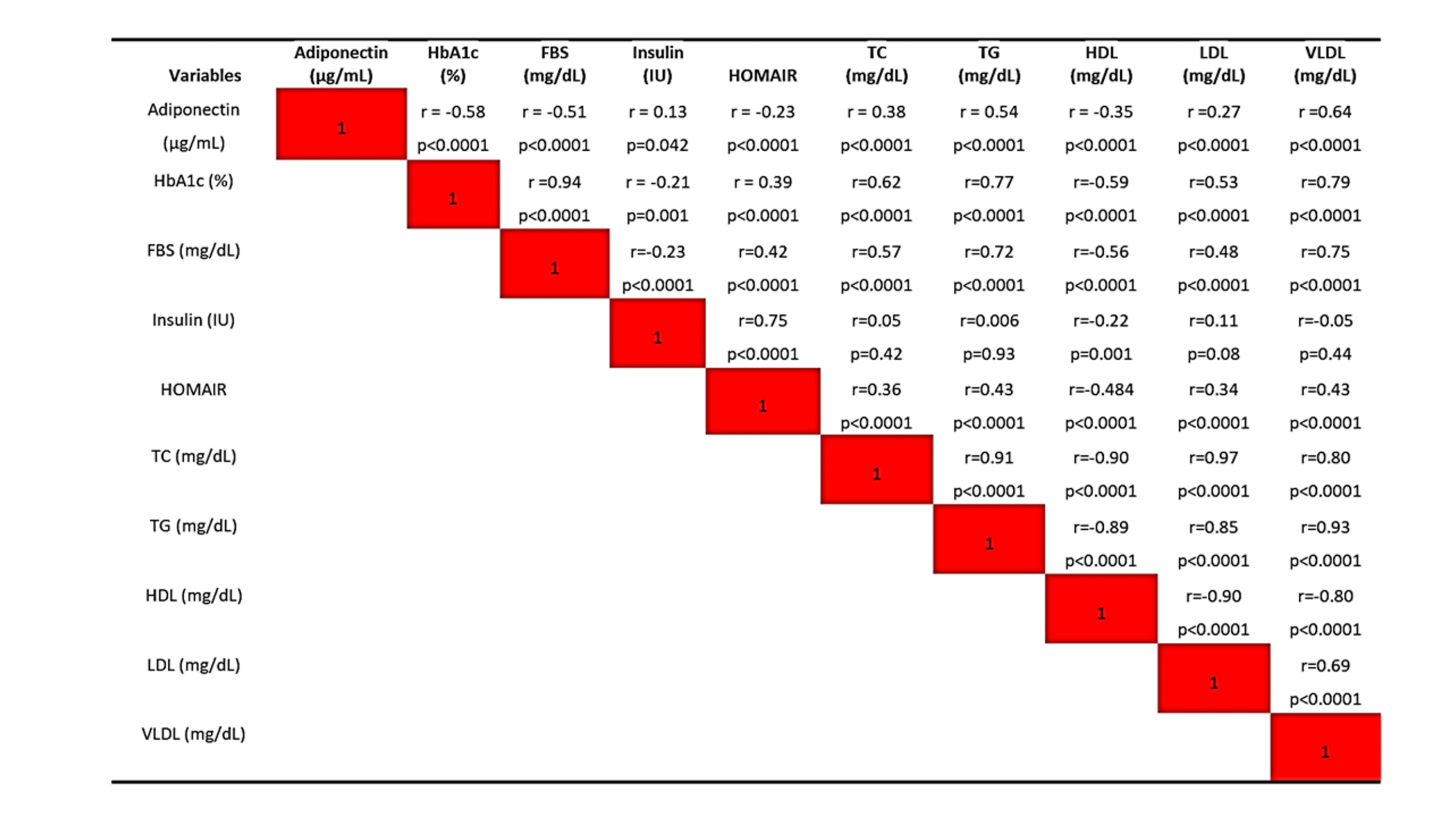
Correlation of adiponectin with the diabetic marker and lipid profile HbA1c: Glycated hemoglobin, FBS: Fasting blood sugar, HOMA-IR: Homeostatic Model Assessment for Insulin Resistance, TC: Total cholesterol, TG: Triglyceride, HDL: High-density lipoprotein, LDL: Low-density lipoprotein, VLDL: Very low-density lipoprotein. The Pearson correlation coefficient was used to see the association between the two variables. *p-value <0.05 was considered statistically significant.

Adiponectin showed a significant (p<0.001 for all) strong negative correlation with HbA1c (r=-0.58,) and FBS (r=-0.51,) respectively. Adiponectin was positively correlated with TC (r=0.38) and negatively correlated with HDL (r=-0.35). There was a positive correlation between adiponectin levels and insulin (r=0.13, p=0.042), and a negative correlation between adiponectin levels and HOMA-IR (r=-0.23, p<0.001) (Figure 1).

The correlation of adiponectin with anthropometric markers

**Figure 2:**
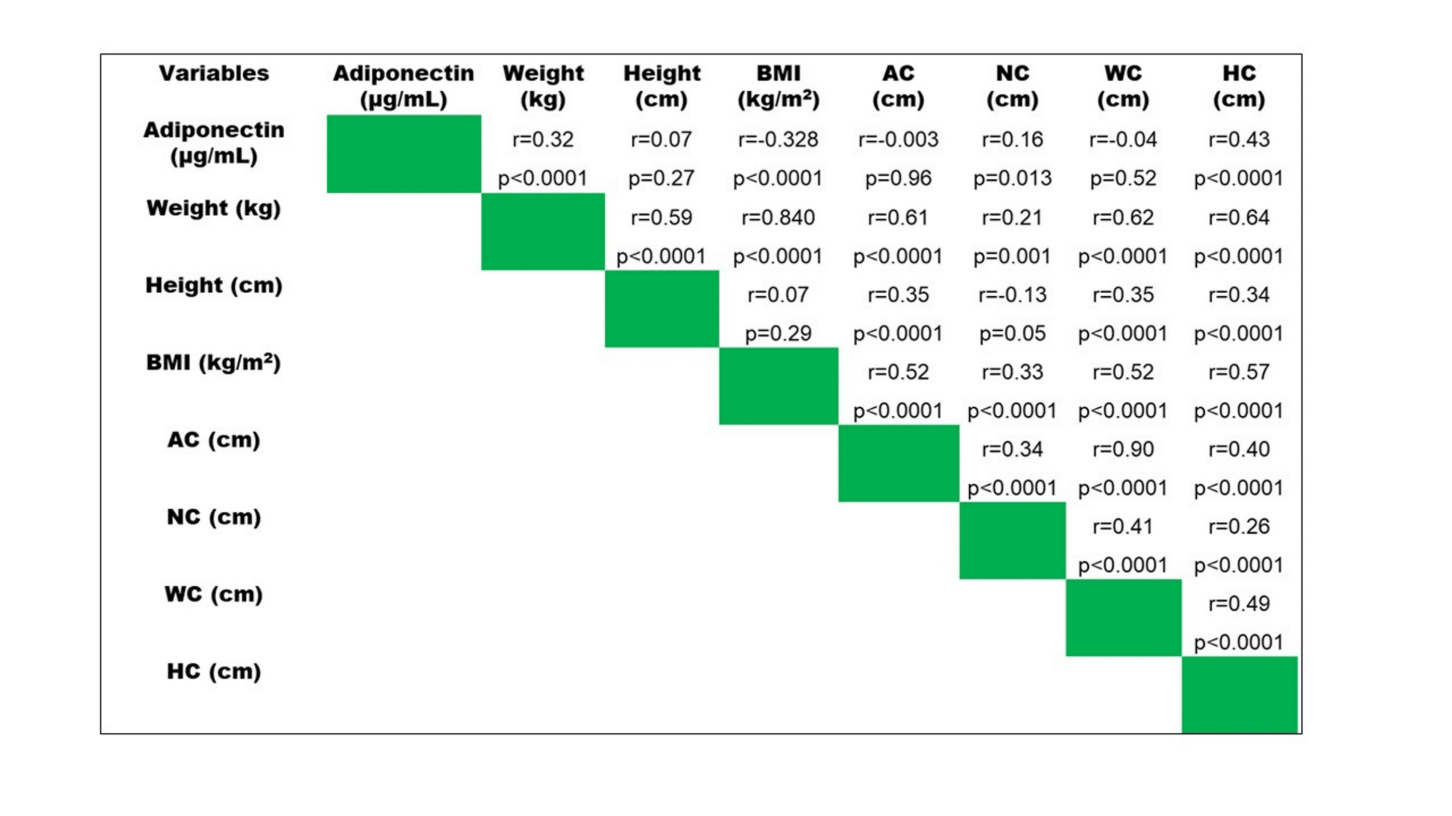
Correlation of adiponectin with anthropometric markers BMI: Body mass index, AC: Abdominal circumference, NC: Neck circumference, HC: Height circumference. The Pearson correlation coefficient was used to see the association between the two variables. *p-value <0.05 was considered statistically significant.

The study demonstrated a significant negative correlation (r=-0.33, p<0.001) between adiponectin levels and BMI, suggesting that low adiponectin levels are related to high BMI. Adiponectin showed a significant but very weak correlation with neck circumference (r=0.16, p=0.013) and HC (r=0.43, p<0.001) (Figure 2).

## Discussion

T2DM is a complex metabolic disorder characterized by reduced insulin production and resistance. Individuals with T2DM are at high risk of developing obesity, dyslipidemia, hypertension, and chronic inflammation [14]. Adipocytes release various cytokines, including adiponectin, which plays a role in protecting against metabolic and cardiovascular disorders. Adiponectin has anti-inflammatory properties and is involved in lipid metabolism and atherogenesis [15]. It increases insulin sensitivity and inhibits inflammatory cytokines such as TNF-α, IL-6, and interferon-γ expression [16]. Studies indicate that adiponectin levels are reduced in obesity and diabetes [8,9].

This study explored the role of adiponectin in T2DM and its association with insulin resistance and anthropometric markers. Our findings demonstrated that non-significant difference in age and gender between the T2DM and control groups, indicating that the groups were an adequate match. The T2DM group exhibited significantly elevated glycemic parameters (FBS, HbA1c, and HOMA-IR), and cardiovascular and anthropometric profiles of the T2DM group showed higher systolic and diastolic blood pressure compared to controls, consistent with findings from previous studies Burchill et al. found that T2DM patients have significantly higher levels of FBS, HbA1c, and HOMA-IR compared to non-diabetic individuals, with elevated HbA1c correlating strongly with increased cardiovascular risk [17]. Smith et al. further confirmed these findings, showing that these elevated parameters are associated with reduced insulin sensitivity and impaired β-cell function [18]. This indicates that as insulin resistance grows, glucose uptake by cells diminishes, leading to higher blood glucose levels and consequently higher FBS and HbA1c. Similarly, Lee et al. demonstrated that elevated glycemic parameters in T2DM are linked to long-term health complications, including cardiovascular diseases and neuropathy [19]. The mechanisms behind these elevations involve chronic hyperglycemia due to insulin resistance, where cells become less responsive to insulin, and β-cell dysfunction, which impairs insulin production. Elevated HOMA-IR values further reflect this imbalance, highlighting the extent of insulin resistance.

Studies have shown that T2DM is associated with significantly higher systolic and diastolic BP. A meta-analysis demonstrated that T2DM patients have elevated BP levels, with an increased risk of hypertension due to factors such as insulin resistance and endothelial dysfunction [20]. A cross-sectional study revealed that higher BP in T2DM is strongly correlated with HbA1c, relating increased blood glucose levels with increased hypertension [21]. Similarly, Kim et al. reviewed the impact of T2DM on BP and highlighted that elevated BP in T2DM patients significantly increases cardiovascular risk [22]. Insulin resistance leads to sodium retention and fluid overload, endothelial dysfunction impairing vessel dilation, and increased vascular stiffness due to chronic hyperglycemia.

Our results revealed significantly lower adiponectin levels in T2DM patients and demonstrated an association between adiponectin levels and hypertension. This is consistent with studies that have shown reduced adiponectin levels in hypertensive individuals compared to normotensive participants [23,24]. This association remained significant even after adjusting for potential confounding factors [23].

The study demonstrated that adiponectin had a significant negative correlation with HbA1c and FBS. On the other hand, adiponectin was positively correlated with TC and negatively correlated with HDL. Adiponectin may play a role in HDL metabolism. Lower adiponectin levels could impair the processes that contribute to HDL formation or increase its catabolism, resulting in lower HDL levels despite elevated TC. HbA1c showed a positive correlation with insulin and a negative correlation with HDL cholesterol. Furthermore, adiponectin levels were positively correlated with insulin and negatively correlated with HOMA-IR. A negative correlation between adiponectin levels and BMI was also observed. Our findings reveal significantly lower levels of adiponectin in patients with T2DM. This reduction in adiponectin is associated with HbA1c, as evidenced by significant negative correlations with HbA1c and FBS. Moreover, adiponectin was negatively correlated with HOMA-IR. Also, a negative correlation between adiponectin levels and BMI was observed. These findings are consistent with studies by Kang et al. [25], Chen et al. [26], and Liu et al. [27], which demonstrated lower adiponectin levels in T2DM patients and their correlations with various metabolic markers. Role of adiponectin in enhancing insulin sensitivity and regulating glucose and lipid metabolism. Lower adiponectin levels contribute to insulin resistance and altered lipid profiles, while higher adiponectin is associated with a lower BMI. Adiponectin interacts primarily with its receptors, AdipoR1 and AdipoR2, in muscle and liver tissues. It enhances insulin sensitivity by activating AMP-activated protein kinase (AMPK), promoting glucose uptake and fatty acid oxidation, and improving lipid metabolism through increased fatty acid oxidation and reduced lipogenesis. Additionally, adiponectin exhibits anti-inflammatory effects by lowering pro-inflammatory cytokines (e.g., TNF-α, IL-6) that hinder insulin signaling, while promoting anti-inflammatory pathways. It also enhances insulin signaling by increasing phosphorylation of IRSs and activating the phosphoinositide 3-kinase (PI3K) pathway, which is essential for glucose uptake [28].

The study has several limitations, including its case-control design, which restricts causal inferences and long-term observations. The sample size may be insufficient for generalizability, and while adjustments were made for various confounders, unmeasured factors such as medication use, diet, and genetic differences could still influence the results. The accuracy of measurements for adiponectin and other metabolic markers depends on the methods used, and variability in these measurements might affect the findings. Additionally, the lack of long-term follow-up limits the ability to assess how changes in adiponectin levels impact T2DM progression and complications. The study's findings may also be less applicable if the sample lacks ethnic and demographic diversity.

## Conclusions

This study highlighted adiponectin levels are lower in patients with T2DM. This decrease in adiponectin is strongly associated with poorer glycemic control, as shown by higher HbA1c, fasting blood glucose levels, and increased insulin resistance. Additionally, adiponectin levels correlate negatively with BMI and positively with TC. These findings suggest that adiponectin may be a valuable biomarker for assessing diabetes risk and metabolic disorders in T2DM.
